# A cross-sectional assessment of symptom burden among patients with advanced cervical cancer

**DOI:** 10.1186/s12904-021-00883-3

**Published:** 2021-12-18

**Authors:** Tolcha Kebebew, Azwihangwisi Helen Mavhandu-Mudzusi, Annah Mosalo

**Affiliations:** 1 Student at University of South Africa, Ethiopia Learning Centre, Addis Ababa, Ethiopia; 2 Department of Health Studies, University of South Africa, Pretoria, South Africa

**Keywords:** Advanced cervical cancer, Cross-sectional assessment, Symptom burden, IPOS, Palliative care, Ethiopia

## Abstract

**Background:**

The increasing burden of chronic non-communicable diseases in developing countries is driving attention to palliative care services. Identification of disease-specific symptoms of concern and their prevalence will guide designing, monitoring, and evaluating palliative care programmes. This study assessed the burden of symptoms and problems among patients with advanced cervical cancer.

**Methods:**

This research followed a cross-sectional study design to quantitatively review the symptom burden among patients diagnosed with advanced cervical cancer attending treatment at Tikur Anbessa Specialised Hospital, Addis Ababa, Ethiopia from January to July 2019. Symptoms were assessed using a patient-reported, seven-day recall Integrated Palliative Care Outcome Scale (IPOS) version III. Frequency, median and mean scores with a standard deviation were used in the descriptive analysis whereas t-test and one-way analysis of variance were used for comparisons.

**Results:**

There were 385 patients with advanced cervical cancer, stage IIB-IVB, successfully interviewed. The median age was 50 years, the majority were illiterate (63.1%) and in marital union (62.3%). Over 50% of the patients experienced pain, weakness, poor appetite, constipation, limited mobility, and dry mouth. The burdens of emotional symptoms such as patient anxiety, family anxiety, and patient depression were also prevalent at 79.7%, 82.3%, and 47.0%, respectively. Patients who are illiterate, at a higher stage of the disease, not currently in marriage, and who received palliative radiotherapy bear a higher symptom burden.

**Conclusion:**

Patients with advanced cervical cancer bear a high symptom burden. Early initiation of palliative care is recommended to alleviate the concerning symptoms, and to improve patients’ quality of life.

## Background

The burden of chronic and non-communicable diseases increases as countries go through an epidemiologic transition [1]. There is insufficient documentation of the prevalence of non-communicable diseases in Ethiopia; however, studies such as Global Observatory of Cancer (GLOBACAN) estimate a high prevalence of cancer [2–4]. The incidence and mortality rate of cervical cancer was the highest in Sub-Saharan African countries with an estimated 34.8 cases and 22.5 deaths per 100,000 population in 2012 [3]. In the Eastern African region, the number is higher, with an incidence rate of 40.1 and a death rate of 30.0 per 100,000 population in 2018 [4]. Breast cancer and cervical cancer are the leading causes of mortality and morbidity among women in Ethiopia [5]. Coupled with an increased proportion of aged people, as the life expectancy has grown from 48 years in 1990 to 66 years in 2015 in Ethiopia [6], chronic diseases pose a greater demand for palliative care services [7, 8].

Palliative care is comprehensive care provided to patients with life-threatening disease conditions to improve the quality of life of the patients and their families [9]. It includes pain control, symptom management, and emotional, socio-economic, psychological and spiritual support, whilst providing the patients with dignity and peace at the end-of-life [9]. In most chronic and life-threatening diseases, the prognosis of cervical cancer after advanced treatment might be poor. Therefore, early initiation of palliative care will help patients and their families as well as the health care workers to avoid ineffective but costly treatments [10].

Assessment and evaluation of palliative care needs can be measured at different levels throughout the care process including the structure, the process, and outcome [11]. However, the most important indicator of palliative care is the outcome status, measured through symptoms and quality-of-life attributes. In the past, having a standardised tool to measure palliative care needs and outcome of the care and support interventions have been a challenge. However, recently there have been different measurement scales developed [11–14]. The Integrated Palliative Care Outcome Scale (IPOS) is one of the most common tools designed to measure the palliative care status [12].

Studies on cervical cancer-specific patient outcomes are lacking in Ethiopia. The outcome measures and the symptom burden estimates could help to design targeted palliative care interventions and to guide the monitoring and evaluation of disease-specific palliative care and support programmes. This study evaluated the burden of symptoms and problems, using the IPOS, and identified the most common symptoms among patients with advanced cervical cancer attending radiotherapy in Addis Ababa, Ethiopia.

## Methods

This study utilised a cross-sectional design using a quantitative approach to assess the burden of the symptoms and problems. Findings of this study were compiled and reported following the “Strengthening the Reporting of Observational Studies in Epidemiology (STROBE)” guidelines [15].

Interviews were conducted with patients diagnosed with advanced cervical cancer, stages IIB-IVB, attending treatment at the Radiotherapy Centre of the Tikur Anbessa Specialised Hospital in Addis Ababa, Ethiopia, from January to June 2019. The hospital is the only oncology centre in the country that provides comprehensive and specialised cancer treatment and care to patients referred from health facilities across the country.

The sample size was determined using the calculation formula for single population proportion studies. Using a proportion of 50%, a 95% confidence level and level of precision of 5%, the sample size (n) was 385 using the formulae: [Z_α/2_/E]^2^pq, where Z_α/2_ is 1.96, proportion (p) and q (1-p) were 0.5 each, and the level of precision (E) was 0.05. Considering an additional 5% for non-response rate, the final sample size was 404.

Participants included in this study were selected by the following criteria: histologically confirmed diagnosis of cervical cancer, stage IIB-IVB according to the International Federation for Gynaecology and Obstetrics (FIGO) [16], age above 18 years, those who were conscious, stable and able to communicate during the interview, and patients who gave consent to participate in the study. After identifying the stage of the disease from patient cards, through the help of hospital nurses, eligible patients were identified each day and included in the study. Whenever there were more than three eligible patients per data collector for each day, we used the simple random sampling technique to select the participants.

The principal researcher, assisted by trained hospital nurses, collected the data using a structured and pre-tested questionnaire that included the seven-day recall IPOS version III to assess the symptom burden. The IPOS scale is composed of items used to measure pain and other symptoms. It is a recommended item used to measure the physical, psychological and mental status and needs among people affected by severe and chronic diseases, including cancer [17, 18]. The IPOS has been adapted and tested in different countries and languages [18–22]; and for different types of diseases including cancer [22], heart failure [23, 24], renal diseases [21], and dementia [25].

Data entry and cleaning were done using CS Pro 7.1 software. The data were then transferred to Stata 12® for analysis. A description of all dependent and independent variables was conducted using frequency, percentage, mean, median, range, or standard deviation, and presented in tables and graphs. The aggregate IPOS scores were compared among different variables using t-test and one-way analysis of variance.

The Research Ethics Committee in the Department of Health Studies, University of South Africa (UNISA), and the Institutional Review Board of the College of Health Sciences, Addis Ababa University granted approval for this study. The Radiotherapy Center of Tikur Anbessa Specialised Hospital also reviewed and approved the study protocol. Each study participant received information on the objectives of the study and the data collection procedures and gave informed consent before the interview. The participants were also provided with participant information sheet. Names and personal identifiers  were kept separate from the actual data and reports to maintain confidentiality.

## Results

### Patient characteristics

A total of 385 patients with advanced cervical cancer were successfully interviewed. The age ranged from 20 to 80 years, with a median of 50 years (Table 1). About 98% (*n* = 376) were patients aged 35 years or more. Nearly one-third (*n* = 138) were among the age group of 35 to 49 years, another one-third (*n* = 125) among the age group of 50 to 60 years, and the rest were more than 60 years of age.

**Table 1:** Socio-demographic and treatment information of patients with advanced cervical cancer, TASH, Ethiopia, 2019 (*n* = 385)

Patient characteristics	Number	Percentage
*Age (Years)*		
Below 50	147	38.2
50 and above	238	61.8
*Educational status*		
Illiterate	243	63.1
Literate	142	36.9
*Marital status*		
Currently in marriage	240	62.3
Currently not in marriage^a^	145	37.7
*Occupation*		
No job	123	32.0
Housewife	81	21.0
Farming	72	18.7
Others^b^	109	28.3
*Average monthly income (in USD)*		
Below 50.00	324	84.2
50.00 or more	61	15.8
*Cancer stage*		
IIB	90	23.4
III (A or B)	105	27.3
IVA	162	42.1
IVB	28	7.3
Treatment modality^c^		
Surgery	39	10.1
Chemotherapy	135	35.1
Therapeutic radiotherapy	139	36.1
Palliative radiotherapy	216	56.1

*TASH* Tikur Anbessa Specialized Hospital, *USD* United States Dollar

^a^includes single or dissolved marriage; ^b^Others include retired, employees, petty traders and daily labourers; ^c^multiple responses possible

Most of the study participants (243, 63.1%) were illiterate, while 119 (30.9%) attended up to secondary education. In total, 240 (62%) were married while 97 (25.2%) and 40 (10.4%) were widowed and divorced, respectively. One hundred and eleven (28.8%) study participants were unemployed at the time of the study, 84 (21.8%) were housewives, 72 (18.7%) were farmers, and 44 (11.4%) were petty traders; government or private employees constituted less than 10% (*n* = 34). The median monthly income was 812.00 Birr (equivalent to 27.07 USD), while only 35 (9.1%) of the participants had a monthly income of 3000.00 Birr (100.00 USD) or higher.

All the study participants had a confirmed diagnosis of advanced cervical cancer, FIGO stage IIB-IVB. Most of the participants (162, 42.1%) were at the stage of IVA followed by 105 (27.3%) at stage III, 90 (23.4%) were at stage IIB, and 28 (7.3%) were at stage IVB. The majority of the study participants (340, 88.3%) had treatment with either curative (therapeutic) or palliative radiotherapy, 139 (36.1%) were treated with chemotherapy, while 39 (10.1%) had undergone surgery (Table 1).

### Symptom burden

#### Main symptoms of concern

**Fig. 1:**
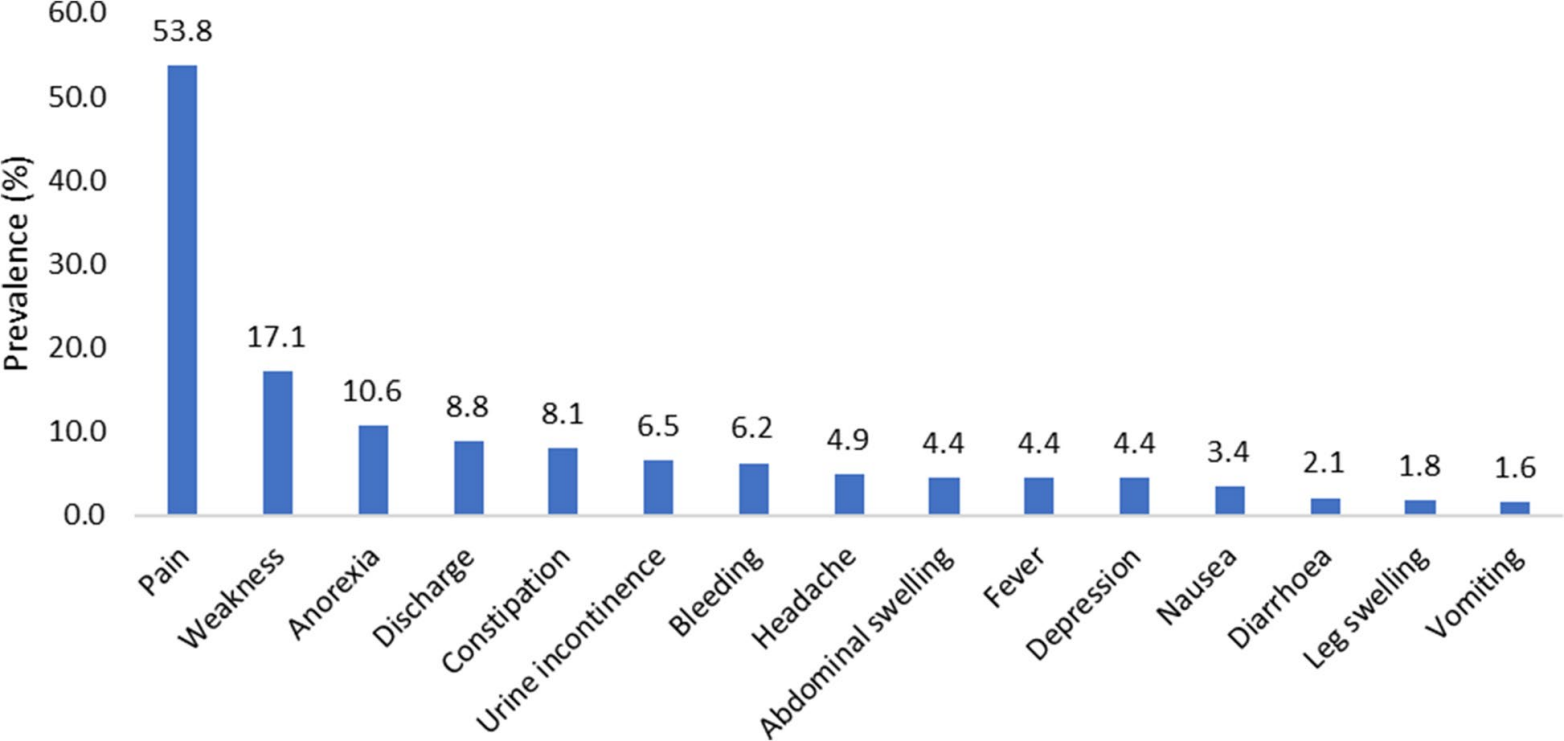
Chief symptoms of concern among patients with advanced cervical cancer in Ethiopia, 2019

The top five symptoms of concern a week before the interviews were pain, among 207 (53.8%), followed by weakness (66, 17.1%), anorexia (41, 10.6%), vaginal discharge (34, 8.8%) and constipation (31, 8.1%), (Fig. 1); other chief presenting symptoms were urine incontinence, vaginal bleeding, headache, abdominal swelling, fever, depression, nausea, diarrhoea, leg swelling, and vomiting.

#### Physical symptoms

**Fig. 2:**
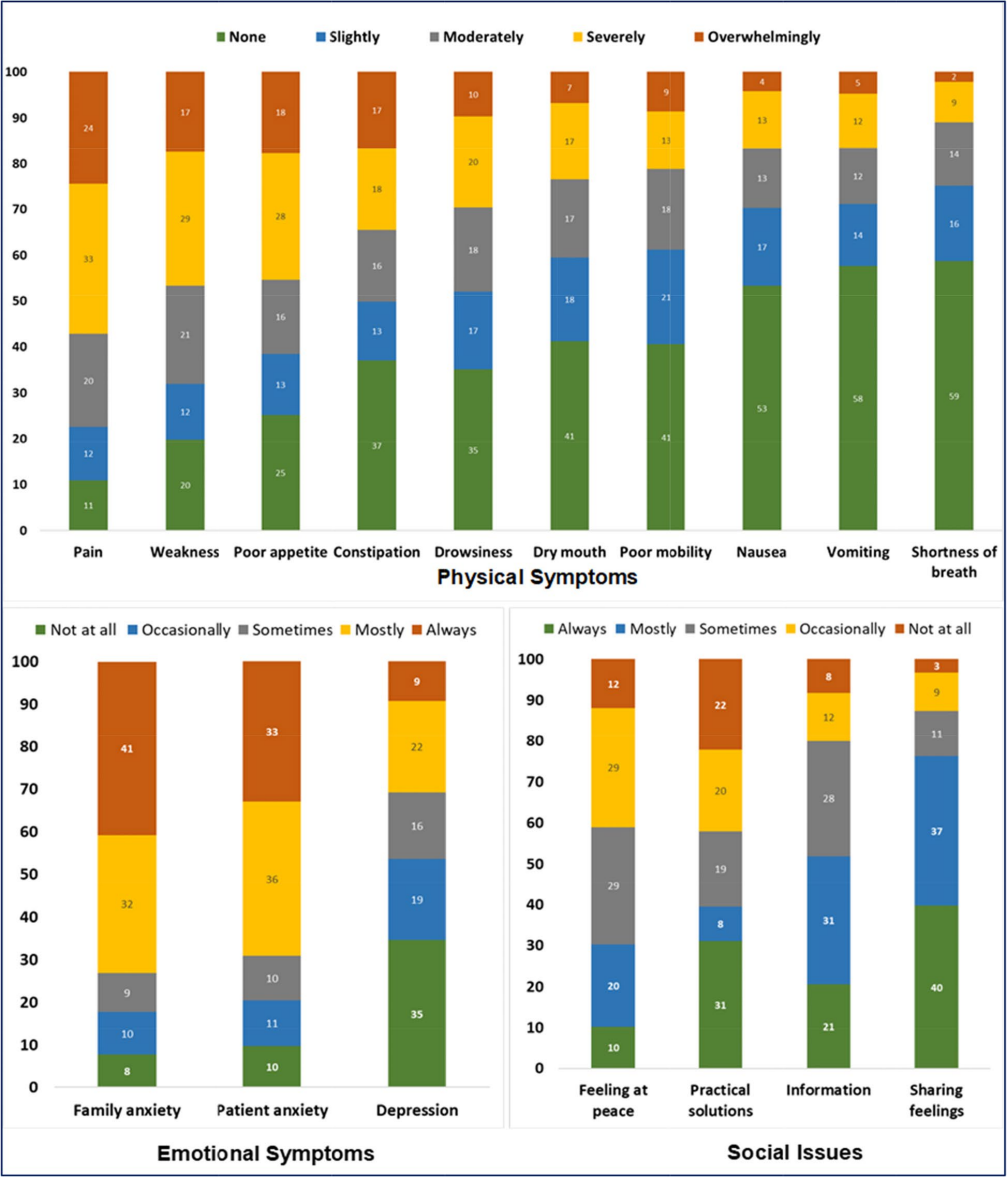
Prevalence (percentage) of IPOS symptoms and problems among patients with advanced cervical cancer in Ethiopia, 2019

The majority (362, 94.0%) of the participants experienced at least one of the IPOS symptoms. The average overall IPOS physical symptom score was 14.9 out of 40, with a standard deviation of 9.0. This showed that the participants were moderately affected, on average, with the 10 common IPOS symptoms. Symptoms that affected at least half of the participants were pain, weakness, poor appetite, drowsiness, constipation, poor mobility, and dry mouth (Table 2 and Fig. 2). Pain was prevalent among 298 (77.4%) of the patients, affecting them moderately to overwhelmingly in the week before the data collection, whereas, 126 (32.7%) and 94 (24.4%) were severely and overwhelmingly affected by pain, respectively. Weakness was also severely (112, 29.1%) and overwhelmingly (67, 17.4%) affecting the patients. The third most prevalent symptom, affecting two-thirds of the patients (232, 62.3%) was poor appetite. It affected 104 (27.0%) severely and 67 (17.8%) overwhelmingly. Table 2 and Fig. 2 display the details of the IPOS symptoms that affected the patients a week before the interview.

**Table 2:** IPOS symptoms among patients with advanced cervical cancer, TASH, Ethiopia, 2019

*Scale, Sub-scales and Items*	*Prevalence*^a^	*Mean*	*SD*
*Physical symptom*^b^			
Pain (*n* = 385)	*77.4*	*2.5*	*1.3*
Weakness/no energy (*n* = 384)	68.1	2.1	1.4
Poor appetite (*n* = 377)	62.3	2.0	1.5
Constipation (*n* = 383)	50.4	1.6	1.5
Drowsiness (*n* = 382)	48.3	1.5	1.4
Dry mouth (*n* = 380)	41.3	1.3	1.3
Poor mobility (*n* = 382)	39.2	1.3	1.3
Nausea (*n* = 384)	29.9	1.0	1.2
Vomiting (*n* = 385)	28.8	0.9	1.3
Shortness of breath (*n* = 381)	25.7	0.8	1.1
*Aggregate score (out of 40, n = 358)*		*14.9*	*9.0*
*Emotional symptoms*^c^			
Family anxiety (*n* = 383)	82.3	2.9	1.3
Patient anxiety (*n* = 383)	79.7	2.7	1.3
Depression (*n* = 380)	47.0	1.5	1.4
Felt at peace^d^ (*n* = 383)	69.7	2.1	1.2
*Aggregate score (out of 16, n = 380)*		*9.3*	*3.7*
*Social & practical conditions*^d^			
Shared feelings (*n* = 383)	23.8	1.0	1.0
Had adequate information (*n* = 383)	48.3	1.6	1.2
Practical problems fully addressed (*n* = 383)	60.8	1.9	1.6
*Aggregate score (out of 12, n = 382)*		*4.5*	*2.7*
*Total score (out of 68, n = 352)*		***28.6***	***12.1***

*IPOS* Integrated Palliative Care Outcome Scale, *TASH* Tikur Anbessa Specialized Hospital, *SD* Standard Deviation

^a^Prevalence (%) is defined as symptoms reported as moderate, severe, or devastating, or problems persisting sometimes, most of the time or always. ^b^Response options: 0=None, 1=Slightly, 2=Moderately, 3=Severely, 4=Overwhelmingly. ^c^Response options: 0=Not at all, 1=Occasionally, 2=Sometimes, 3=Mostly, 4=Always. ^d^Response options: 0=Always, 1=Mostly, 2=Sometimes, 3=Occasionally, 4=Not at all

#### Emotional symptoms

Anxiety and depression among patients and anxiety among the families were the emotional symptoms of the IPOS tool. A high proportion of the patients and their families experienced anxiety, while patient depression was relatively lower. More than three-quarters (305, 79.7%) of the study participants experienced anxiety always, mostly, or sometimes; only 37 (9.7%) did not feel anxious at all. About one-third (131, 34.5%) of the patients did not feel depressed at all, while 176 (47.0%) felt depressed sometimes, mostly, or always. About one-third (117, 30.8%) felt depressed most of the time or all the time. The prevalence of family anxiety was also very high; 315 (82.3%) felt anxious sometimes, most of the time, or all the time. A significant proportion, (267, 69.7%), did not feel at peace all the time, mostly or sometimes, while 116 (30.3%) felt at peace always or most of the time.

#### Social conditions and practical problems

Social and practical problems among the study participants were prevalent. Getting required information among the patients was a concern sometimes, always, or most of the time in 185 (80.0%) of the patients. Nearly two-thirds of the participants (233, 60.8%) felt that their practical problems such as personal and financial issues were not appropriately addressed, and a significant proportion (161, 42.0%) felt that these problems were hardly addressed. Nearly one quarter (91, 23.8%) of the participants did not usually share their feelings with their families or friends.

##### Other symptoms

Other symptoms that affected the patients were vaginal discharge, vaginal bleeding, urine incontinence, headache, fever, abdominal swelling, diarrhoea, insomnia, and leg swelling, affecting a proportion ranging from 10.4 to 2.1%, in respective order (Table 3). These symptoms have affected the patients moderately, severely, or extremely. Vaginal bleeding was a concern among 40 (10.4%) mainly overwhelmingly (32, 8.3%) and severely (6, 1.6%), whereas, both vaginal discharge and urine incontinence affected 26 (6.8%) of patients.

**Table 3:** Other physical symptoms among patients with advanced cervical cancer, TASH, Ethiopia, 2019

*Symptom*	*P*^a^	*Mean*	*SD*	*Frequency, Number (Percentage)*				
				***None***	***Slightly***	***Moder-*** ***ately***	***Severely***	***Overwh-*** ***elmingly***
				***0***	***1***	***2***	***3***	***4***
Vaginal discharge	10.4	0.4	1.2	345 (89.6)	–	2 (0.5)	6 (1.6)	32 (8.3)
Vaginal bleeding	6.8	0.3	1.0	359 (93.2)	–	2 (0.5)	2 (0.5)	22 (5.7)
Urine incontinence	6.8	0.3	1.0	359 (93.2)	–	–	1 (0.3)	25 (6.5)
Headache	6.0	0.2	0.9	362 (94.0)	–	3 (0.8)	3 (0.8)	17 (4.4)
Fever	5.2	0.2	0.9	365 (94.8)	–	–	1 (0.3)	19 (4.9)
Abdominal swelling	4.9	0.2	0.8	366 (95.1)	–	–	2 (0.5)	17 (4.4)
Diarrhoea	2.1	0.1	0.5	377 (97.9)	–	2 (0.5)	–	6 (1.6)
Insomnia	2.1	0.1	0.5	377 (97.9)	–	1 (0.3)	1 (0.3)	6 (1.6)
Leg swelling	2.1	0.1	0.4	377 (97.9)	–	2 (0.5)	5 (1.3)	1 (0.3)

*TASH* Tikur Anbessa Specialized Hospital, *SD* Standard Deviation

^a^P refers to prevalence (%) that is defined as symptoms reported as moderate, severe, or overwhelming

##### IPOS aggregate scores

The aggregate scores of the physical, mental and social problems were calculated. The mean aggregate physical symptom score was 14.9 ± 9.0 out of 40, while that of mental symptoms was 9.3 ± 3.7 out of 16. The mean social problems score was 4.5 ± 2.7 out of 12, while the mean overall aggregate IPOS score out of 68 was 28.6 ± 12.1 (Table 2).

The mean physical, emotional, social and overall scores were compared among different categories of socio-demographic and disease and treatment-related variables (Table 4). Patients aged below 50 years expressed a higher score of emotional problems (*p* = 0.022) whereas illiterate patients had higher physical, social and overall problem scores (*p* = 0.002, *p* = 0.020 and *p* = 0.004, respectively).

**Table 4:** IPOS scores & associated factor among patients with advanced cervical cancer, TASH, Ethiopia, 2019

Socio-demographic and treatment information	Physical (0–40)	Emotional (0–20)	Social (0–12)	Overall (0–68)
Age (years)				
Below 50	*14.86*	9.81*	*4.61*	*29.50*
50 and above	14.92	8.92	4.41	27.97
Educational status				
Illiterate	16.03**	*9.40*	4.73*	30.03**
Literate	12.98	9.03	4.07	26.11
Marital status				
In marriage	*14.62*	8.85**	*4.41*	27.70*
Not in marriage	15.34	9.94	4.62	29.96
Occupation				
No job	*14.91*	*9.43*	*4.69*	*28.99*
Housewife	15.16	8.89	4.43	28.12
Farming	16.15	9.46	5.01	30.29
Others^a^	13.84	9.21	4.18	27.23
Monthly income				
Below 50.00 USD	*15.21*	*9.20*	4.70**	*29.00*
50.00 USD or more	13.20	9.59	3.36	26.17
Cancer stage				
IIB	12.30**	8.07**	*4.49*	24.91**
III (A or B)	15.24	9.02	4.52	28.68
IVA	15.76	9.87	4.38	29.86
IVB	16.86	10.61	4.93	32.39
Surgery				
No	14.78	9.40*	4.52	28.61
Yes	15.94	8.03	4.23	28.23
Chemotherapy				
No	14.64	9.43	4.40	28.34
Yes	15.37	8.96	4.66	28.98
Therapeutic radiotherapy				
No	16.39**	9.86**	4.43	30.65
Yes	12.28	8.19	4.58	24.87
Palliative radiotherapy				
No	12.53**	8.26**	4.51	25.24**
Yes	16.77	10.06	4.47	31.22
*Aggregate*	***14.90***	***9.26***	***4.49***	***28.57***

*IPOS* Integrated Palliative Care Outcome Scale, *TASH* Tikur Anbessa Specialized Hospital, *USD* United States Dollar

^a^Others include retired, employees, petty traders and daily labourers; * Significantly different at 0.05 using t-test or one-way analysis of variance; ** Significantly different at 0.01 using t-test or one-way analysis of variance

Patients who were in marriage had significantly lower emotional problems (*p* = 0.006), while patients with monthly income below USD 50.00 had shown a significantly higher burden of social problems compared to those with higher income (*p* = 0.000). Patients with FIGO stage IIB had lower physical, emotional and overall problem scores (*p* < 0.01) compared to those at stage III and stage IV. Surgery was significantly associated with emotional problems (*p* = 0.030); a significantly lower score was observed among those who underwent surgery. Patients who took treatment with therapeutic radiotherapy had significantly lower physical and emotional problems, whereas palliative radiotherapy showed a reverse effect (*p* < 0.01). The overall IPOS score was also significantly higher among those who took palliative radiotherapy (*p* = 0.000).

## Discussion

This study identified the burden of symptoms among patients with advanced cervical cancer using the seven-day recall patient version III of the IPOS. Pain, weakness, and anorexia were the most concerning symptoms 7 days before the interview. Pain, poor appetite, patient anxiety, family anxiety, and poor mobility were the most prevalent symptoms. This study also identified symptoms not included in the IPOS tool, such as vaginal discharge, vaginal bleeding, and urine incontinence, which could be relevant during the development of cervical cancer-specific palliative care interventions.

Pain was the most common symptom, experienced by 77.4% of the participants and the most prevalent concern in the same period. This finding is comparable to a previous study in Ethiopia which documented that 80% of non-communicable diseases experience moderate to severe pain [26]. Other studies in Ethiopia, among patients with chronic diseases, also reported that over three-quarters of them experienced pain [27, 28]. The prevalence of pain was very high compared to a study done amongst Portuguese cancer patients [19], which reported a prevalence of 42%; the pain prevalence was also higher than studies done in other countries: in Japan among cancer patients (66%), [22], in the UK and Germany among cancer patients (62%), [18], and in the Czech Republic among patients with chronic diseases including cancer (52%), [29]. Another study among patients with heart failure reported a lower prevalence of 40%, [24]. A higher pain prevalence could be due to poor pain control practices in Ethiopian health care settings [30, 31], patient attitude [32], or socio-economic factors [33, 34]. In Ethiopia, there was documentation of the usage of weak analgesics, such as paracetamol and ibuprofen, instead of morphine and other opioids for the treatment of severe pain [35]. A study conducted among cancer patients in Ethiopia showed that about two-thirds of them received inadequate pain management; only 19% received opioids such as codeine and morphine for the management of severe pain [28, 35].

Weakness was the second most common symptom with a prevalence of 68.1%. The symptom was less common in comparison to patients with cancer in Europe and Asia [18, 22, 29], but higher than the prevalence among Portuguese cancer patients [19] and patients with heart failure [23, 24]. Poor appetite, constipation, nausea, and vomiting were more prevalent compared to studies in Europe, [18, 19, 24, 29], whereas it was less common than the prevalence in Japanese cancer patients [22]. Symptoms such as drowsiness, dry mouth, lack of mobility, and shortness of break were less common [18, 19, 22, 24, 29].

Emotional symptoms, including patient anxiety, family anxiety, depression, and failure to feel at peace were common among the study participants. Anxiety among the patients was common at a prevalence of 79.7%, while family anxiety was even higher at 82.3%. Patient anxiety was higher than the findings among cancer patients in different countries [18, 19, 24, 29], but less common than the findings in Japanese cancer patients [22]. Compared to the findings from these countries, there were comparable prevalence of family anxiety, depression, and failure to feel at peace among patients with chronic diseases.

Social interaction and solutions to practical problems such as financial and personal problems were very low. Only 23.8% of the patients shared their feelings with their families or friends. Similarly, only 48.3% felt they received as much information as they needed, while about 60% of the patients felt they had their practical problems addressed. Compared to these reports, findings in the studies referenced above show that a higher proportion of patients shared their feelings, had adequate information, and practical problems such as financial constraints solved [18, 19, 22, 24, 29].

There was a higher burden of emotional symptoms observed among women in the reproductive age group and those unmarried; this could relate to sexual activity and childbearing attitudes. Lack of support from the spouse or family could also be a contributing factor. Illiterate patients had a higher burden of physical and social problems; a lower educational status could hinder the utilisation of treatment and palliative care services as well as the education and counselling. Treatment with palliative radiotherapy and a higher stage of the disease were associated with a higher burden of physical, emotional and overall problem scores. Palliative radiotherapy, given at a late stage, added to the unmanaged side effects, could have contributed to a higher symptom burden. An explorative research could be necessary to study why palliative radiotherapy could not reduce the burden of physical and emotional symptoms.

## Conclusion

This study has identified the symptoms of importance among patients with advanced cervical cancer. It also provided important symptoms in addition to the existing list in the IPOS tool. The prevalence of symptoms of concern, such as pain, weakness, and anorexia, were high in comparison to references from various countries. The burden of symptoms and problems were higher among women of childbearing age, unmarried, illiterate, those who took palliative radiotherapy, and patients with low socioeconomic status. Patients who took surgery and curative radiotherapy had a lower burden. Identification of cervical cancer-specific symptoms and concerns could help to design and implement targeted palliative care interventions. We recommend early detection and alleviation of these symptoms and concerns to improve the quality of life of the patients.

## Data Availability

This study is part principal author’s PhD thesis at UNISA. The dataset collected and analysed in this study is available from the corresponding author upon reasonable request and after UNISA grants permission.

## Abbreviations

FIGO: International Federation of Gynaecology and Obstetrics
GLOBOCAN: Global Observatory of Cancer
IPOS: Integrated Palliative Care Outcome Scale
STROBE: Strengthening the Reporting of Observational Studies in Epidemiology
UNISA: University of South Africa
WHO: World Health Organization
